# Fluorescent reporters for markerless genomic integration in *Staphylococcus aureus*

**DOI:** 10.1038/srep43889

**Published:** 2017-03-07

**Authors:** Nienke W. M. de Jong, Thijs van der Horst, Jos A. G. van Strijp, Reindert Nijland

**Affiliations:** 1 Medical Microbiology, University Medical Center Utrecht, Utrecht, The Netherlands; 2 Laboratory of Phytopathology, Wageningen University, Wageningen, The Netherlands

## Abstract

We present integration vectors for *Staphylococcus aureus* encoding the fluorescent reporters mAmetrine, CFP, sGFP, YFP, mCherry and mKate. The expression is driven either from the *sarA*-P1 promoter or from any other promoter of choice. The reporter can be inserted markerless in the chromosome of a wide range of *S. aureus* strains. The integration site chosen does not disrupt any open reading frame, provides good expression, and has no detectable effect on the strains physiology. As an intermediate construct, we present a set of replicating plasmids containing the same fluorescent reporters. Also in these reporter plasmids the *sarA*-P1 promoter can be replaced by any other promoter of interest for expression studies. Cassettes from the replication plasmids can be readily swapped with the integration vector. With these constructs it becomes possible to monitor reporters of separate fluorescent wavelengths simultaneously.

*Staphylococcus aureus* is one of the most problematic pathogens for human health, able to cause skin and soft tissue infections, as well as serious invasive diseases such as sepsis, endocarditis, osteomyelitis, toxic shock syndrome and pneumonia^1^. Tools to analyse host-pathogen interaction and gene expressions in *S. aureus* are still in short supply. In the last years several very useful constructs have been made which aid in the study of *S. aureus* using fluorescent proteins, using either replicating plasmids^2^,^3^, or integrating the reporter into the genome with the aid of a selectable resistance marker^4^. Also plasmid systems to integrate the fluorescent reporters at the locus of the original genes has been described, using the presence of a *bursa aurealis* transposon in the genome of the target strains created for and available through the Nebraska Transposon Mutant Library^5^. However, as this system requires the previous insertion of the transposon, it is not readily applicable to other strains of *S. aureus*.

The use of fluorescence reporters is clear in the field of microscopy and flow cytometry analysis. However, also in plate readers with the capability to measure fluorescence both growth (constitutive promoter) as well as specific promoter activity can be measured, such as demonstrated in ref. 6.

In this study, we build upon the constructs already available to the *S. aureus* research community^2^,^3^,^4^. We created constitutively expressed fluorescent reporters CFP, GFP, YFP and multiple red variants (DsRED, mCherry, mKate2). Further, we introduce a new fluorescent reporter already in use in eukaryotic systems but so far absent from prokaryotic toolboxes, mAmetrine^7^. This latter protein is excited at a much shorter wavelength, but fluoresces at a wavelength similar to GFP. The expression of these reporters is controlled by the *sarA-*P1 promoter and the expression cassette is integrated in the genome of the *S. aureus* strains by homologous recombination. As an intermediate, but similarly useful tool, we produced replicating plasmids containing all mentioned reporter genes, and wherein the *sarA-*P1 promoter, which is flanked by restriction sites, can be replaced by any other promoter of interest for expression studies. With these constructs it becomes possible to study multiple promoter activities in different fluorescent colours. For example, it is possible to transform a GFP containing plasmid under control of a promoter of your interest, in bacteria constitutively expressing mAmetrine or CFP from its chromosomal location, and analyse promoter activity compared to bacterial growth in conditions where OD cannot be measured reliably due to the turbidity of the medium used or in (confocal) microscopy when non-fluorescent cells are very difficult to spot.

## Materials and Methods

### General procedures, plasmids, bacterial strains and media

All plasmids and strains used in this study are listed in Table 1. All plasmids were transformed into competent *E. coli* DC10B or *E.coli* Top10F′. Correct plasmids isolated from *E. coli* DC10B were directly transformed to *S. aureus* Newman, MW2 and USA300 strains by electroporation as described^8^. If the plasmid was isolated from *E.coli* Top10 F′ it was first introduced in *S. aureus* RN4220, re-isolated and subsequently introduced in the target *S. aureus* strain. *E. coli* was grown in LB broth or agar. *S. aureus* was grown in Todd-Hewitt agar or broth. When growing in liquid cultures, both *E. coli* and *S. aureus* were grown in 50 ml tubes with maximum 10 ml of growth medium, shaken at 300 rpm at 37 °C. Ampicillin concentrations for *E. coli* were 100 μg/ml and chloramphenicol concentrations for *S. aureus* were 10 μg/ml. All restriction enzymes were purchased from New England Biolabs and were used according to the manufacturer’s instructions. PCR was performed using Phusion DNA polymerase (Finnzymes) or PWO polymerase (Roche) for cloning, and using Taq-ready mix (Fermentas) for colony checking. Primers were obtained from Integrated DNA Technologies (IDT, Leuven, Belgium) and sequencing reactions were performed using BigDye Terminator V3.1 (Life Technologies) and analysed at the Central Sequence Facility at UMC Utrecht, or at Macrogen (Macrogen Easy-seq). PCR purifications were performed using a QIAquick PCR Purification Kit (Qiagen). Ligations were performed using a T4 DNA Ligase (Roche Applied Science) according to the manufacturer’s instructions. Initial fluorescence expression was analysed using an ImageQuant LAS4010 (GE Health Care) or Leica TSC SP5 inverted microscope equipped with a HCX PL APO 40/0.85 objective (Leica Microsystems), with the correct excitation and emissions spectra for the different fluorescent colours (Table 2).

### Design of *S. aureus* codon optimized mAmetrine coding sequence

The fluorescent protein mAmetrine has a very large stokes-shift^7^, with an excitation maximum at 423 nm and emission maximum at 525 nm. The original coding sequence available in the literature had a high GC-content and is used in eukaryotes, and we assumed this to be less compatible with expression in low GC gram-positive bacteria such as *S. aureus*. Therefore, its codon usage was optimized using the software Gene Designer 2.0 (DNA 2.0, USA) and *S. aureus* MW2 genome sequence as a reference for codon usage. A G-block of 750 bp containing the newly designed coding sequence with a GC-content of 29% and upstream RBS and spacing to start of the reporter gene identical to pCM29 was ordered from IDT (IDT, Leuven, Belgium). Additional restriction sites were added by PCR (primers in Table 3), and this PCR product was cloned into a KpnI, EcoRI digested pCM29 as described below. The nucleotide sequence of the codon optimized mAmetrine gene is deposited at GenBank: KX759016.

### DsRED, mKate2, mCherry, YFP & CFP and fluorescent reporters in plasmid pCM29

DsRed.T3(DNT), mKate2, mCherry, YFP (venus) and CFP (Cerulean) coding genes were amplified by PCR from several templates (templates and primers are presented in Table 3). For amplification of mCherry and mKate2 the forward primer was designed to include XmaI, KpnI and Bsu36I restriction sites, a RBS, and spacing to start of the gene encoding the reporter protein identical to the one present in pCM29 (Table 3). For mKate2 genomic DNA of *S. pneumoniae* strain MK119^9^ was used as a template. This strain contains a *S. pneumoniae* codon optimized gene encoding mKate2 C-terminally fused to a histone protein. To create a single gene also the ATG start codon was added to the primer sequence (Table 3).

To create the mAmetrine, CFP, YFP, and DsRED expressing vectors, the pCM29 plasmid and PCR amplified reporter DNA were digested with KpnI and EcoRI. To create the mKate2 and mCherry constructs, pCM29 and PCR amplified reporter DNA were digested XmaI-EcoRI. After digestion, pCM29 was dephosphorylated, separated on an agarose gel, and the plasmid backbone was isolated from gel. The digested reporters were ligated into pCM29-backbone. The resulting plasmids were named pTH1 (DsRED), pTH2 (CFP), pTH3 (YFP), pRN10 (mKate2), pRN11 (mCherry), pRN12 (mAmetrine). All plasmids were transformed to competent *E. coli* DC10B and, after initial selection based on expected fluorescence spectrum of the colonies using the ImageQuant LAS4010 (GE Healthcare), checked for correct ligation and absence of mutations by restriction analysis and sequencing.

### Creating integration plasmid pJB38-NWMN29-30

By PCR a chromosomal fragment of *S. aureus* Newman DNA (GenBank: AP009351.1) consisting of the region spanning from the nucleotides 40813-43668 containing the 3′ part of NWMN_0029 and the 3′ part of NWMN_0030 (NWMN29-30) was amplified using primers NWMN_0029-FW_EcoRI and NWMN_0030-RV_KpnI (Table 3). The amplified sequence was introduced into plasmid pJB38 by digesting pJB38 and the PCR product with EcoRI and KpnI followed by ligation. The plasmid was transformed in competent *E. coli* DC10B and analysed by restriction analysis and sequencing.

### Addition of fluorescent reporters into pJB38-NWMN29-30

**Figure 1:**
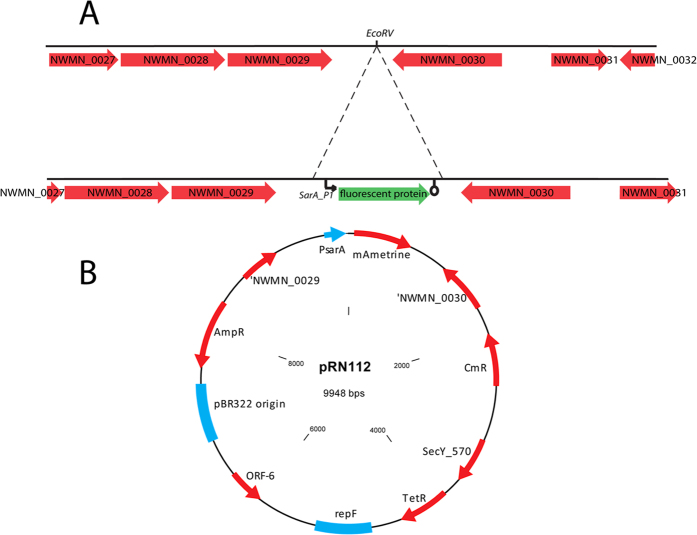
Genomic region of *S. aureus* strain Newman where the fluorescent contrusts were integrated. (**A**) Schematic overview of the insertion site for the fluorescent protein expression construct in the chromosome of *S. aureus* Newman (NC_009641.1). The construct is introduced by a double cross over event between the genes NWMN0029 and NWMN0030. (**B**) Plasmid pRN1112 used for integration by double cross over of PsarA_P1-mAmetrineA construct in the chromosome *of S. aureus* strains. CmR = chloramphenicol resistance gene; TetR: Tetracycline resistance gene; AmpR = Ampicillin resistance gene.

The fluorescent reporter expression cassettes present in the replicating shuttle vectors described above were amplified by PCR, and simultaneously a transcription terminator was added. The sequence encoding the transcription terminator was obtained from vector pMUTIN2^10^ and build into the primer pCM29_reporter_RV+ term. pJB38-NWMN29-30 was digested with EcoRV (the EcoRV site is present in the Newman chromosomal DNA fragment) and dephosphorylated using shrimp alkaline phosphatase (SAP, Thermo Scientific). The PCR products were phosphorylated with polynucleotide kinase (PNK, NEB) and ligated into the plasmid. The plasmids were transformed in *E. coli* DC10B and initial selection was based on expected fluorescence spectrum of the colonies using the ImageQuant LAS4000. Fluorescent colonies were further analysed by plasmid isolation and restriction analysis. Plasmids containing the expression cassette in the desired orientation (expression cassette integrated in the direction of gene NWMN_0029) were checked by sequencing, and were designated pTH100 (sGFP); pTH101 (DsRed); pTH102 (CFP); pTH103 (YFP); pRN110 (mKate); pRN111 (mCherry) and pRN112 (mAmetrine). As an example, a map of plasmid pRN112 is given in Fig. 1B. The new plasmids described here are available through Addgene, and we provide their properties and Addgene plasmids numbers in Suppl. Table 2.

### Chromosomal integration of fluorescent reporters in *S. aureus*

The fluorescent protein expression cassettes were integrated markerless into the *S. aureus* chromosome between genes NWMN_0029 and NWMN_0030 (or homologs in the other strains, see Fig. 1A and Suppl. Table 1) by homologous recombination as described previously^11^ with some adaptations. Shortly, plasmids pTH100, pTH101; pTH102; pTH103; pTH104, pRN110, pRN111 and pRN112 were introduced into the *S. aureus* strains Newman, MW2 and USA300 by electroporation and plated on TH agar containing chloramphenicol, and incubated overnight at 30 °C. Fluorescent colonies were picked to a fresh plate and incubated at 45 °C overnight to select for integration of the plasmid into the chromosome. Large colonies were picked (single-recombinants) and re-streaked on TH+Cm plates and grown at 45 °C overnight. Single colonies were picked to Todd-Hewitt medium without antibiotics and incubated at 30 °C, 250 rpm overnight. Cultures were diluted 1:1000, cultured during 7 hours (during day time), re-diluted, grown for 16 hours (overnight) etc., resulting in 5–7 dilution-culturing cycles, to allow for double cross over events to occur. The final culture was plated on Todd-Hewitt agar containing 100ng/ml anhydrotetracycline and incubated O/N at 37 °C to select for double cross over mutants. The anhydrotetracyclin induces the expression of the counter-selection marker present on the pJB38 derivative plasmids^5^. Single colonies were plated on Todd-Hewitt plates with (AB+) and without (AB−) antibiotics and grown at 37 °C. Colonies that were fluorescent and not able to grow on the AB+ plate (double-recombinants) were analysed by PCR for correct integration.

### Measuring growth and fluorescence in a plate reader

**Figure 2:**
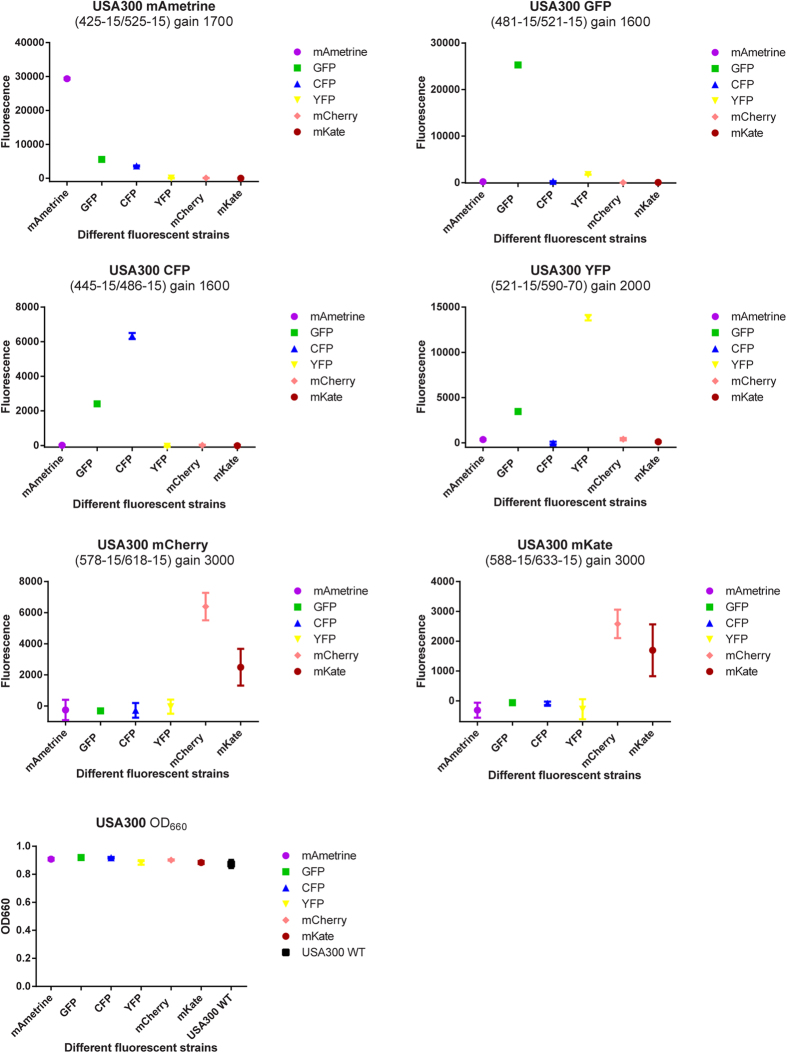
Spectral separation and intensity of all fluorescent constructs obtained by integration in the chromosome of *S. aureus* strains USA300. Data points show mean +/− SD is shown of 4 technical replicates.

Bacterial growth (optical density) and fluorescence were measured. Bacteria were diluted to an OD_660_ of 0.01 and 150 μl was transferred to selected wells of a clear 96 well flat bottom polystyrene tissue culture plate (Greiner). The plate was incubated directly in a Fluostar Omega or Clariostar plate reader (BMG labtech) at 37 °C with constant double orbital shaking (400 rpm) in between measurements. Both the optical density at 660 nm and fluorescence of mAmetrine, CFP, GFP, YFP DsRED, mKate, mCherry were measured every 10 minutes for each well. The signal from 4 identical wells was averaged and corrected for blank wells containing only medium. Settings for the wavelengths measured using the Fluostar and Clariostar plate readers are given in Table 2, Fig. 2 and suppl. Fig. 1.

### mAmetrine excitation and emission spectra measurement

The excitation and emission spectra were recorded using a BMG labtech Clariostar plate reader. 100 μl of an overnight culture of *S. aureus* USA300 containing plasmid pRN12 were loaded in the wells of a flat bottom 96 well plate. Excitation was scanned using settings: excitation scan: 339.0–484.0; stepwidth 1.0 nm, bandwidth 10 nm; emission wavelength at 519 nm, bandwidth 16 nm. Emission was scanned using settings: excitation wavelength 401 nm, bandwidth 16 nm; Emission scan 447.0–624.0 nm; stepwidth 1.0 nm, bandwidth 8 nm. To correct for autofluorescence, a similar overnight culture of wildtype *S. aureus* USA300 was taken along, and the spectra from this scan were used as blank.

### Microscopy

Microscopic image acquisition of fluorescent bacteria was performed using a Leica TSC SP5 inverted microscope equipped with a HCX PL APO 40 × 0.85 CORR CS and HCX PL APO 63x/1.40–0.60 oil immersion (Leica Microsystems, The Netherlands). The microscope was encased in a dark environment chamber with temperature control. The cells and bacteria were monitored in brightfield, using wide field epifluorescence, or in confocal mode setting. Using the correct settings, it was possible to obtain an image where four fluorophores could be separately identified in one sample. The separate channels were combined using Leica LAS AF software.

## Results and Discussions

### Plasmids containing expression cassettes of various fluorescent proteins

**Figure 3:**
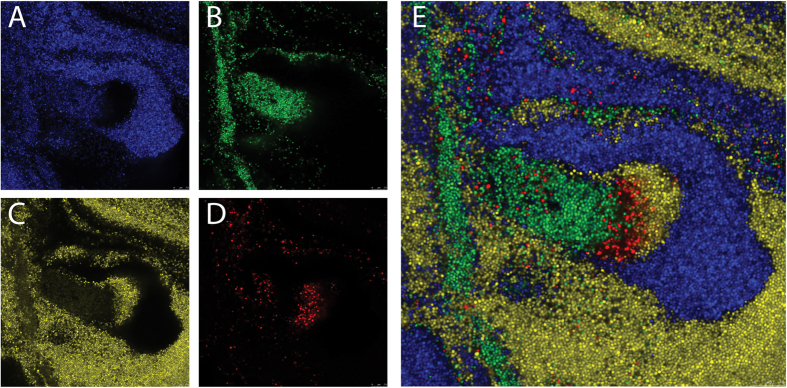
Simultaneous imaging of a mix of 4 separate *S. aureus* strains expressing fluorescent proteins from replicative plasmids using confocal microscopy. Acquisition settings: (**A**) CFP: excitation laser 458 nm, detection 465–502 nm; (**B**) GFP: excitation laser 476 nm, detection 500–510 nm; (**C**) YFP: excitation laser 514 nm, detection 525–600 nm; (**D**) DsRed: excitation laser 543 nm, detection 598–709 nm. (**E**) Composite image of (**A–D**).

pCM29 was chosen as starting vector for building all other reporter plasmids, since it is a stable plasmid with replicates both in *E. coli* and *S. aureus* and contains sGFP under the control of *sarA*-P1 promoter^12^. By exchanging sGFP with DsRED, mCherry, mKate2, CFP, YFP and mAmetrine we created pTH1, pRN10, pRN11, pTH2, pTH3 and pRN12 respectively with the same backbone as pCM29. In these plasmids multiple unique restriction sites are present flanking both sides of the *sarA-*P1 promoter, allowing for easy exchange of this promoter with any other promoter to study its expression. In their present form with the *sarA*-P1 promoter these plasmids give a very strong fluorescent signal, and it is possible to precisely separate the cells expressing each of the four different fluorescent proteins in one sample (Fig. 3). The fluorescent signal from the DsRed construct was sometimes strong, but unfortunately the expression of this specific fluorescent protein appeared unstable compared to the other fluorescent proteins, and a large population of cells rapidly lost DsRed expression after continued subculturing (not shown).

### mAmetrine excitation and emission spectra

**Figure 4:**
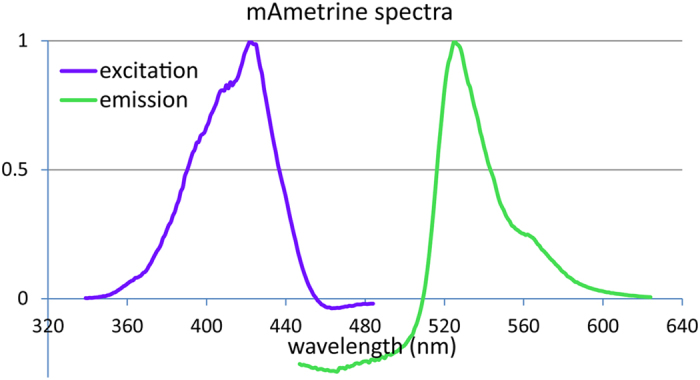
Excitation and emission spectra for mAmetrine when expressed in the cytosol of *S. aureus* USA300.

To determine the excitation and emission spectra of mAmetrine when expressed in the cytosol of *S. aureus*, we measured these spectra (Fig. 4). A maximum excitation of mAmetrine was observed at 422 nm, and a maximum emission at 525 nm, similar to the reported values for mAmetrine in the literature^7^. As *S. aureus* shows autofluorescence, particularly when excited with blue light, this correction of the spectra using a non-fluorescent strain resulted in negative numbers for the emission spectrum below 510 nm.

### Selection of site for markerless genomic integration

The stable integration of a reporter gene in the bacterial genome has several benefits over expression from a replicating plasmid. However, integration also has some drawbacks, notable a lower signal due to its single presence per copy of the chromosome and possible pleiotropic effects on the disrupted genes. To reduce these drawbacks as much as possible, our integration site was selected based on the following criteria: to minimize downstream effects of the integration, the reporter cassette should not be integrated inside an operon or in a promoter sequence. Preferably it should be integrated at the 3′ end of both open reading frames between which it will be located. Furthermore, the locus should be relatively close to the chromosomal origin of replication. In exponentially growing cells, chromosome division is an almost continuous process resulting in a higher copy number of genes close to the origin of replication compared to genes close to the terminus^13^. It is therefore advantageous to integrate a reporter construct close to the origin as this will result in more copies of the construct and therefore a brighter signal. To minimize the impact of integration on the adjacent genomic regions, we selected a region with two open reading frames that are transcribed in opposing directions, their stop codons facing each other. The reporters are then integrated into their shared downstream region, in such a way that no nucleotides are replaced, but simply the reporter cassette is added, as such influencing only the spacing between the two open reading frames.

The site chosen to integrate the reporter cassette is located between genes NWMN_0029 and NWMN_0030 (*S. aureus* Newman; NC_009641.1). There is 666 bp spacing between the two open reading frames, and the presence of an endogenous EcoRV recognition site in the region between genes 29 and 30 (EcoRV site 167 bp down 29; 499 bp down 30) in Newman which was not present in the cloning vector pJB38 was of practical benefit (Fig. 1). Gene NWMN_0029 is annotated as a hypothetical protein similar to a pyridine nucleotide-disulfide oxidoreductase, and gene NWMN_0030 is annotated as a tRNA-dihydrouridine synthase.

Finally, the region chosen was highly conserved in the sequenced *S. aureus* strains. In a Blast search against *S. aureus* genomes available at Genbank, the selected region of the Newman chromosome was shown to be identical in USA300, NCTC8325, COL, and has 1 basepair difference in strain RN4220. The lowest similarity encountered of the fragment used for homologous recombination was 99.5% (1794/1803), present in strains MSSA476 and MW2. In the later strain integration was not more problematic than with strain Newman. Note that in the different *S. aureus* strains the gene names and numbers differ. For USA300 the genes are not designated gene numbers 29 and 30, instead gene NWMN_0029 is identical to SAUSA300_0087 (due to the integration of SSC-mec and ACME upstream of the selected site). Suppl. Table 1 lists the corresponding location of the integration site in frequently used *S. aureus* strains.

When going through the genome of *S. aureus* strain Newman another region showed to be adhering the criteria set above, and could be useful for making double labelled strains or stable integration of other genes of interest. This is the genomic region between genes NWMN_0048/NWMN_RS00280 and NWMN_0049/NWMN_RS00285 (63771 bp–64264 bp). The reason not to select this region here was because it is slightly less conserved across most *S. aureus* strains compared to the 29_30 region. It was 100% homologous to strains Newman, Col, USA300, NCTC 8325, there was a little gap in this region in strains MSSA476 and MW2, and a homology below 95% in N315, Mu50.

### Remark on the selected genomic integration site

**Figure 5:**
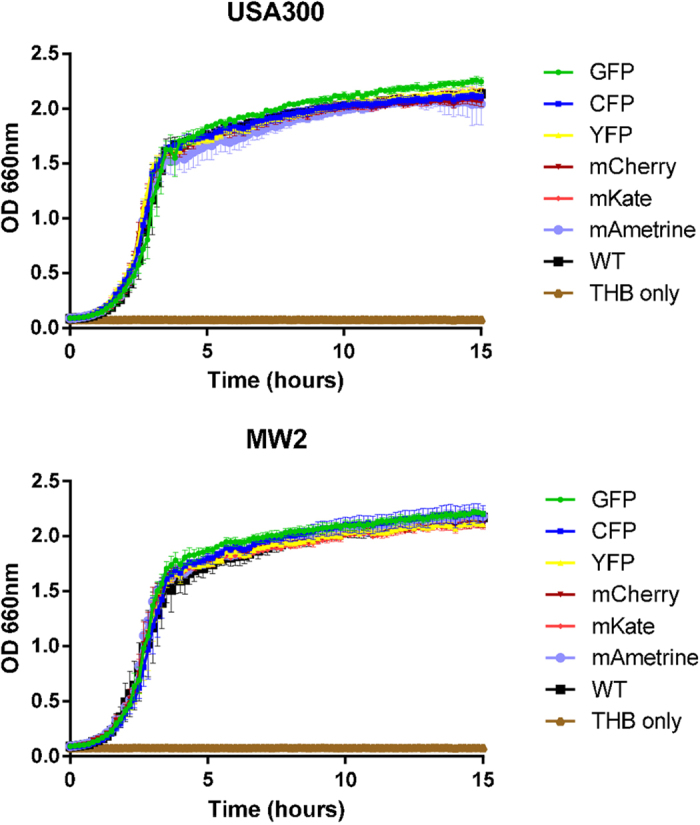
Growth of *S. aureus* strains USA300 and MW2 with chromosomal integration of different fluorescent markers. No effect on growth dynamics of the genomic integration of the various fluorescent constructs is visible.

Recently all bacterial genomes, including those of most *S. aureus* strains, were re-annotated at NCBI, and their locus tags have changed^14^,^15^, see also Suppl. Table 1. In this new annotation, a very small open reading frame is annotated between genes 29 and 30. This open reading frame is annotated to encode USP (universal stress protein). The sequence is starting with an unusual start codon (TTG) and is very short (102 bp). Upon close inspection of the sequence, both a potential RBS (GGAGGTA) is present 17 bp upstream of the start codon, and also a potential promoter binding site (−35 TTTACA, −10 AATTAAAAT), BPROM^16^ is predicted 66 bp upstream of the TTG start codon. The EcoRV site used to insert the fluorescent sequences is 36 bp upstream of the start of the −35 site of this predicted promoter. As a result, the location chosen between the opposing genes NWMN_0029 and NWMN_0030 no longer confirms to all criteria set out above. The insertion site is now situated between genes 30 and the USP-gene, which are in a similar orientation, although not likely to be in an operon. The NARSA transposon insertion database^17^ shows no insertion in homologs of genes 29 (SAUSA300_0087/SAUSA300_RS00450) and SAUSA300_RS00455 (although 29 homolog is not shown in the NARSA database as its sequence contains a premature stop codon in and is therefore annotated as a pseudogene). Insertions were abundantly present in the homolog of gene 30 (SAUSA300_0089/SAUSA300_RS00460). Nevertheless, the relative ease of making insertions and double cross over events indicates that our insertion does not have a strong effect on the fitness of the strain, and also growth is comparable between the control and fluorescent strains (Fig. 5).

### Markerless genomic integration of genes encoding fluorescent proteins

pJB38 is a plasmid with a temperature sensitive replicon for *S. aureus*, which can be used for chromosomal integration^5^. By adding the NWMN29-NWMN30 sequence, pJB38-NWMN29-30 was created as a backbone for targeted integration in the *S. aureus* genome. Markerless insertion of the constructed fluorescent expression cassettes mAmetrine, CFP, sGFP, YFP, mCherry, mKate was efficient, both in stains Newman, MW2 and USA300. Furthermore, the sGFP cassette (pTH100) was successfully inserted in strain SH1000 (Personal communication François Vandenesch). Unfortunately, the DsRED construct was difficult to obtain, and when a clone with the construct integrated in the chromosome was eventually isolated it rapidly lost DsRED expression, likely due to secondary mutations. This again indicated, as seen with the replicative plasmid, that DsRED was a problematic fluorescent protein to express in the *S. aureus* strains used. As an alternative we have created both mCherry and mKate constructs, whose excitation and emission spectra are both at a longer wavelength compared to DsRed, allowing for improved separation of multiple fluorophores in the same sample.

### Stability of the reporters and influence on growth rate

To test whether the newly integrated reporters influenced the growth of *S. aureus*, we have grown both the WT strain and the reporter containing strains in a microplate reader. As is clear from Fig. 5, no differences in growth were detected for both MW2 and USA300. Fluorescence could be detected for all fluorescent proteins using the appropriate excitation and emission setting (Table 2, Fig. 2, suppl. Fig. 1), and during exponential growth a correlation was present between OD_660_ and fluorescence measurements, with the most similarity between optical density and fluorescence signal for GFP, CFP and YFP. The mAmetrine signal also followed the growth curve, but the mKate and mCherry signals are lagging, likely due to lower folding kinetics of these fluorescent proteins^18^. Once cells reach stationary phase the OD_660_ obviously plateaus, but the fluorescence signal continues to accumulate, indicating that the activity of the *SarA*-P1 promoter continues into stationary phase. Our results demonstrate that for the constructed reporter strains measuring fluorescence is a suitable alternative to measuring OD if turbidity due to the presence of eukaryotic cells such as neutrophils will prevent correct OD measurements. Depending on the type of fluorescent protein chosen the signal should be corrected based on the known maturation characteristics of the protein.

### Potential applications of the developed tools

The constructs described here have a broad spectrum of applications in biological research. By using different fluorescent proteins for the specific promoter activity and the genomic integrated reporter, you can differentiate between bacterial growth and promoter activity. This setup will give novel information in a quick and dynamic way. This can be done with any promoter of *S. aureus*, which will greatly enhance research on this harmful bacterium. Alternatively, in a co-infection experiment, one could use different fluorescent chromosomal encoded markers in each strain, which after the experiment can readily be quantified using flow cytometry or, after plating, simply counting the number of blue, green and red fluorescent colonies using a fluorescence imager such as a LAS4010.

The plasmid backbone used for all reporter expression plasmids (from pCM29) contains the open reading frame encoding the Rep-1 protein for rolling circle replication in gram-positive hosts^12^. As such, this plasmid enables replication in several other gram-positive bacteria in addition to *S. aureus*. We have tested *Bacillus subtilis* and *Paenibacillus sp*. Both organisms become brightly fluorescent when transformed with the pCM29 plasmid (data not shown), demonstrating that the collection of replicative plasmids described here is also able to drive high expression of fluorescent proteins from the *sarA-*P1 promoter in other Firmicutes.

## Additional Information

**How to cite this article**: de Jong, N. W. M. *et al*. Fluorescent reporters for markerless genomic integration in *Staphylococcus aureus. Sci. Rep.* **7**, 43889; doi: 10.1038/srep43889 (2017).

**Publisher's note:** Springer Nature remains neutral with regard to jurisdictional claims in published maps and institutional affiliations.

## Supplementary Material

Supplementary Data

## Figures and Tables

**Table 1:** Strains and plasmids used in this study.

Strain/plasmid	Genotype/properties	Reference
*E. coli*		
DC10B	cloning strain, *dcm*-	19
Top10F′	cloning strain	Invitrogen
*S. aureus*		
RN4220	ST8; CC8; chemically mutagenized derivative of 8325–4, transformable with *E. coli* DNA	20
Newman	WT, NC_009641.1	
Newman-mAmetrine	integration of sarA_P1-mAmetrine-Term downstream gene NWMN_0029	This study
Newman-CFP	Integration of sarA_P1-CFP-Term downstream gene NWMN_0029	This study
Newman-sGFP	Integration of sarA_P1-sGFP-Term downstream gene NWMN_0029	This study
Newman-YFP	Integration of sarA_P1-YFP-Term downstream gene NWMN_0029	This study
Newman-mCherry	Integration of sarA_P1-mCherry-Term downstream gene NWMN_0029	This study
Newman-mKate2	Integration of sarA_P1-sKate2-Term downstream gene NWMN_0029	This study
MW2	WT, NC_003923.1	
MW2-CFP	Integration of sarA_P1-CFP-Term downstream gene MW0058	This study
MW2-mAmetrine	Integration of sarA_P1-mAmetrine-Term downstream gene MW0058	This study
MW2-sGFP	Integration of sarA_P1-sGFP-Term downstream gene MW0058	This study
MW2-YFP	Integration of sarA_P1-YFP-Term downstream gene MW0058	This study
MW2-mCherry	Integration of sarA_P1-mCherry-Term downstream gene MW0058	This study
MW2-mKate2	Integration of sarA_P1-sKate2-Term downstream gene MW0058	This study
USA300_FPR3757	WT strain, NC_007793.1	
USA300-mAmetrine	Integration of sarA_P1-mAmetrine-Term downstream pseudogene SAUSA300_0087	This study
USA300-CFP	Integration of sarA_P1-CFP-Term downstream pseudogene SAUSA300_0087	This study
USA300-sGFP	Integration of sarA_P1-sGFP-Term downstream pseudogene SAUSA300_0087	This study
USA300-YFP	Integration of sarA_P1-YFP-Term downstream pseudogene SAUSA300_0087	This study
USA300mCherry	Integration of sarA_P1-mCherry-Term downstream pseudogene SAUSA300_0087	This study
USA300-mKate2	Integration of sarA_P1-sKate2-Term downstream pseudogene SAUSA300_0087	This study
Plasmids		
pCM29	Plasmid for *sarA* P1- sGFP expression	12
pKTEI	Plasmid containing DsRed.T3 (DNT)	21
pJL76	Plasmid containing CFP (Cerulean)	3
pJL77	Plasmid containing YFP (Venus)	3
pJL49	Plasmid containing Pagr-mCherry	3
pTH1	Plasmid for sarA P1- dsRED expression	This study
pTH2	Plasmid for sarA P1- CFP expression	This study
pTH3	Plasmid for sarA P1- YFP expression	This study
pRN10	Plasmid for sarA P1- mKate expression	This study
pRN11	Plasmid for sarA P1- mCherry expression	This study
pRN12	Plasmid for sarA P1- mAmetrine expression	This study
pJB38	Temperature sensitive plasmid for chromosomal integration	5
pJB38-NWMN29-30	pJB38 plasmid containing the Newman genetic region between genes 29 and 30	This study
pTH100	pJB38-NWMN29-30 + SarA_P1-sGFP-Term	This study
pTH101	pJB38-NWMN29-30 + SarA_P1-DsRed-Term	This study
pTH102	pJB38-NWMN29-30 + SarA_P1-CFP-Term	This study
pTH103	pJB38-NWMN29-30 + SarA_P1-YFP-Term	This study
pRN110	pJB38-NWMN29-30 + SarA_P1-mKate2-Term	This study
pRN111	pJB38-NWMN29-30 + SarA_P1-mCherry-Term	This study
pRN112	pJB28-NWMN29-30 + SarA_P1-mAmetrine-Term	This study

**Table 2:** Properties of fluorescent proteins constructed.

Fluorescent protein	Excitation maximum	Emission maximum	LED excitation and filter used in imager	Filters cubes for epifluorescence microscopy	Detection settings in monochromator based plate reader	Detection settings in filter based plate reader: Ex filter/Em filter	Reference
mAmetrine	423	525	N/A	A ex: BP340-380 dichroic 400 em LP425	425-15/525–15	420–10/520EM**	7
CFP (cerulean)	430	475	N/A	CFP ET ex: BP436/20 dichroic 455 em BP480/40	445–15/486–15	420–10/485EX*	3
sGFP	488	511	EpiRGB: Blue/510DF10 GFP	GFP ET ex: BP470/40 dichroic 500 em BP525/50	481–15/524–15	485EX*/520EM**	12
YFP (venus)	515	528	EpiRGB: Blue/510DF10 GFP	YFP ET ex: BP500/20 dichroic 515 em BP535/20	521–15/590–70	485EX*/520EM**	3
mCherry	587	610	EpiRGB: Green/575DF20 Cy3	N21 ex: BP 515–560 dichroic 580 em LP590	578–15/618–15	544EX***/590-bp10	3
mKate2	588	633	EpiRGB: Green/575DF20 Cy3	N21 ex: BP 515–560 dichroic 580 em LP590	588–15/633–15	544EX***/590-bp10	9

All wavelength in nm.

Em = emission; Ex = excitation; BP = bandpass filter; LP: longpass filter,

*bp 475–490 nm; **bp 520–555 nm; ***bp 530–560 nm.

**Table 3:** Overview of the DNA-templates and primers used in this study.

Template	Primer	Orientation	Primer Sequence
pKTE1	DsRED-fw_kpnI++	FW	ATCCCCGGGTACCAGGAGGAAAAACATATGGACAACA
	DsRED-rv	RV	CGCGCCTGAATTCCTACAGGAACAGGTGGT
pJL76	CFP-YFP-fw_kpnI++	FW	ATCCCCGGGTACCTTAGGAGGATGATTATTTATGAGTAAAG
	CFP-YFP-rv-EcoRI	RV	TGACGAATTCTTACTTGTACAGCTCGTCCAT
pJL49	mCherry-FW-KpnI++_RBS	FW	ATCCCCGGGTACCTTAGGAGGATGTATACATATGGTGAGCAAGGGCGAGGAG
	mCherry+Term-rv_EcoRI	RV	TGACGAATTCGCCTGTCACTTTGCTTGATATATGAG
*S.pneumoniae* MK119 chromosomal DNA	mKate-FW_KpnI++_RBS_ATG	FW	ATCCCCGGGTACCTTAGGAGGATGATTATTTATGTCAGAACT-TATCAAGGAAAATATGCACATGAA
	mKate_rv-EcoRI	RV	TGACGAATTCTTAACGGTGTCCCAATTTACTAGG
750 bp IDT-Gblock	mAmetrine gblock FW	FW	ATGTATCGAGCAAGATGCATCGGATCCCCGGGTACCTT-AGGAGGATGATTATTTATGGTTTCAAAAG
	mAmetrine gblock RV	RV	ATAACAATTTCACACAGGAAACAGCTATGACATGATTA-CGAATTCTTATTTATATAATTCATCCATACCTGGTGTAAT
pCM29/other plasmids	pCM_PsarAfw-seq	FW	TTGCATGCCTGCAGGTCGACTCTA
	pCM29-reporter-RV-seq	RV	TTATGCTTCCGGCTCGTATGTTGTGTGG
*S. aureus* Newman chromosomal DNA	NWMN_0029-FW_EcoRI	FW	TCACGAATTCAGTGGCTACATTCGAACATATCAA
	NWMN_0030-RV_KpnI	RV	GACTGGTACCGTAAGGGTTCCGGCTTAAT
All replicative reporter plasmids	pCM29_reporter_RV+ term	RV	ATATCGCGAGCTGCATAAAAAACGCCCGGCGGCAACCG-AGCGTTCTGAATTAACACACAGGAAACAGCTATGACATGATTA
	pCM_reporterFW-nruI	FW	ATATCGCGATTGCATGCCTGCAGGTCGACTCTA
	NWMN29end_FW	FW	TATGTCACTTATCCTTTTGGAAATG
	NMWN30end_RV	RV	CATAATGTGTTGTAAACATTTTTTTTG
	pJB38-fw_seq	FW	AACCTATAAAAATAGGCGTATCA
