# Supplementary material for: Fluorescent reporters for markerless genomic integration in Staphylococcus aureus
Source: Sci Rep. 2017 Mar 7;7:43889. doi: 10.1038/srep43889 (PMC5339689; doi:10.1038/srep43889)
Supplement: Supplementary Data [file srep43889-s1.pdf]

Fluorescent reporters for markerless genomic integration in *Staphylococcus aureus*

Nienke de Jong[a], Thijs van der Horst[a], Jos van Strijp[a] and Reindert Nijland[a,b,*]

a) Medical Microbiology, University Medical Center Utrecht, Utrecht, The Netherlands
b) Laboratory of Phytopathology, Wageningen University, Wageningen, The Netherlands

*) Corresponding author:
Tel: +31 317 4 83427
e-mail: reindert.nijland@wur.nl

Supplemental data

Supplemental table 1: Gene identifiers for commonly used *S. aureus* laboratory strains for genes identical to *S. aureus* Newman gene 29.

S. aureus subsp. aureus strain:	NCBI Reference Sequence + location in chromosome	Locus tag NWMN_0029 homolog<br>Re-annotated locus tag (14)
Newman	NC_009641.1 (40821..42014)	NWMN_0029<br>NWMN_RS00180
MW2	NC_003923.1 (67771..68964)	MW0058<br>MW_RS00305
USA300_FPR3757	NC_007793.1 (95560..96753)	SAUSA300_0087<br>SAUSA300_RS00450
COL	NC_002951.2 (74754..75947)	SACOL0065<br>SACOL_RS00335
NCTC 8325	NC_007795.1 (40821..42014)	SAOUHSC_00037<br>Not re-annotated?
N315	NC_002745.2 (93968..95161)	SA0084<br>SA_RS00575
MSSA476	NC_002953.3 (66478..67671)	SAS0058<br>SAS_RS00285

Supplemental table 2: Availability of plasmids and cloning vectors

plasmid	Genotype/properties	Plasmid + sequence available from
pCM29	Plasmid for *sarA* P1- sGFP expression	Pang. *et al*. Journal of Innate Immunity 2, 546 (2010)
pTH1	Plasmid for *sarA* P1- dsRED expression	Addgene, #84451
pTH2	Plasmid for *sarA* P1- CFP expression	Addgene, #84452
pTH3	Plasmid for *sarA* P1- YFP expression	Addgene, #84453
pRN10	Plasmid for *sarA* P1- mKate expression	Addgene, #84454
pRN11	Plasmid for *sarA* P1- mCherry expression	Addgene, #84455
pRN12	Plasmid for *sarA* P1- mAmetrine expression	Addgene, #84456
pJB38	Temperature sensitive plasmid for chromosomal integration	BEI, https://www.beiresources.org/ http://app1.unmc.edu/fgx/Static/pJB38.txt
pJB38-NWMN2930	pJB38 plasmid containing Newman gene region 29-30	Addgene, #84457
pTH100	pJB38-NWMN29-30 + SarA_P1-sGFP-Term	Addgene, #84458
pTH101	pJB38-NWMN29-30 + SarA_P1-DsRed-Term	Addgene, #84459
pTH102	pJB38-NWMN29-30 + SarA_P1-CFP-Term	Addgene, #84460
pTH103	pJB38-NWMN29-30 + SarA_P1-YFP-Term	Addgene, #84461
pRN110	pJB38-NWMN29-30 + SarA_P1-mKate2-Term	Addgene, #84462
pRN111	pJB38-NWMN29-30 + SarA_P1-mCherry-Term	Addgene, # 84463
pRN112	pJB28-NWMN29-30+ SarA_P1-mAmetrine-Term	Addgene, # 84464

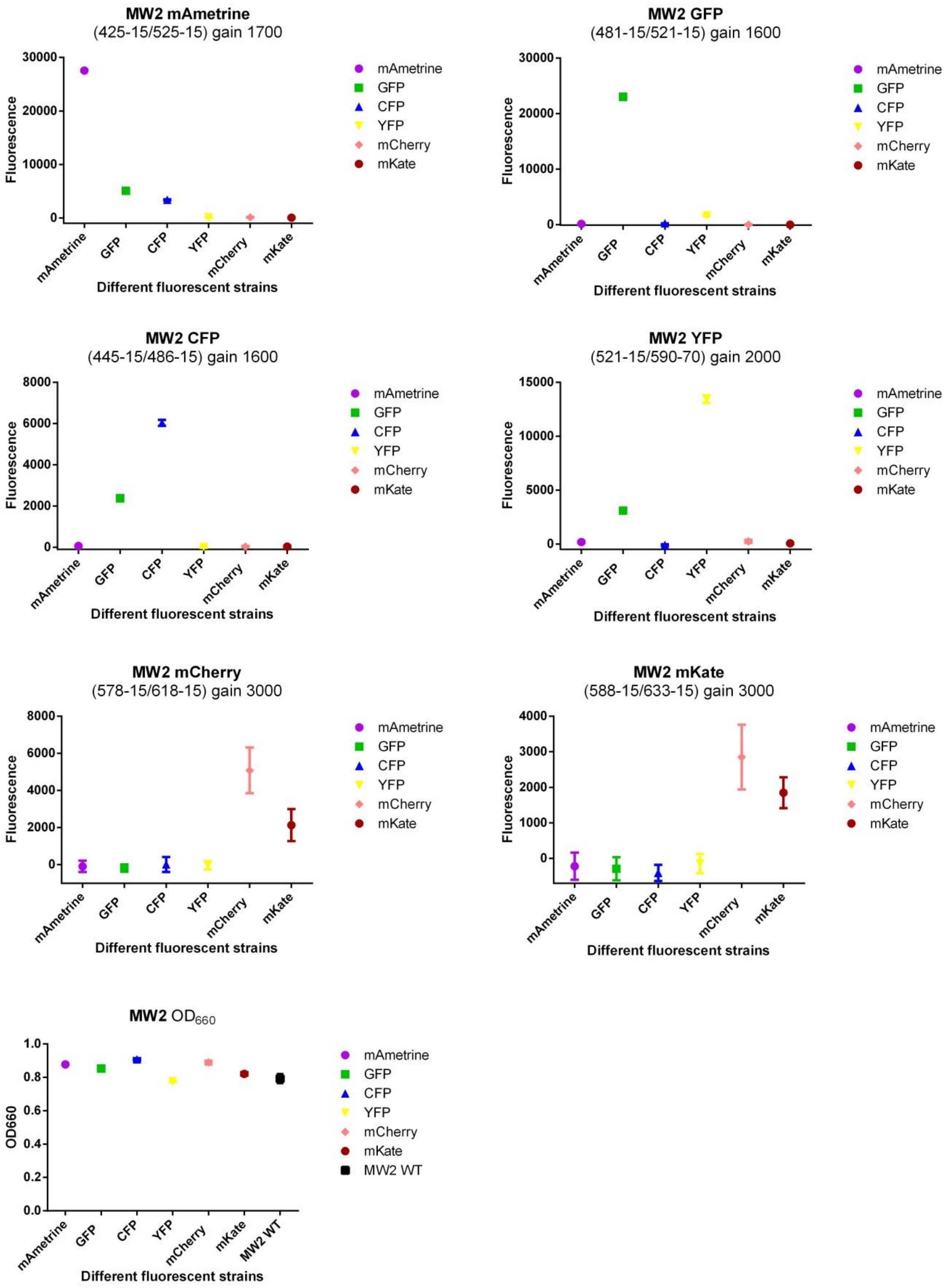

Supplemental fig 1: Spectral separation and intensity of all fluorescent constructs obtained by integration in the chromosome of *S. aureus* strains MW2. Data points show mean +/- SD is shown of 4 technical replicates.
